# Sterol and Sphingoid Glycoconjugates from Microalgae

**DOI:** 10.3390/md16120514

**Published:** 2018-12-17

**Authors:** Valentin A. Stonik, Inna V. Stonik

**Affiliations:** 1 G.B. Elyakov Pacific Institute of Bioorganic Chemistry, Far Eastern Branch, Russian Academy of Sciences, Pr. 100-let Vladivostoku 159, 690022 Vladivostok, Russia; stonik@piboc.dvo.ru; 2 National Scientific Center of Marine Biology, Far Eastern Branch, Russian Academy of Sciences, Palchevskogo Str, 17, 690041 Vladivostok, Russia

**Keywords:** microalgae, sterol glycoconjugates, glycosylceramides, structures, biological activities, functions

## Abstract

Microalgae are well known as primary producers in the hydrosphere. As sources of natural products, microalgae are attracting major attention due to the potential of their practical applications as valuable food constituents, raw material for biofuels, drug candidates, and components of drug delivery systems. This paper presents a short review of a low-molecular-weight steroid and sphingolipid glycoconjugates, with an analysis of the literature on their structures, functions, and bioactivities. The discussed data on sterols and the corresponding glycoconjugates not only demonstrate their structural diversity and properties, but also allow for a better understanding of steroid biogenesis in some echinoderms, mollusks, and other invertebrates which receive these substances from food and possibly from their microalgal symbionts. In another part of this review, the structures and biological functions of sphingolipid glycoconjugates are discussed. Their role in limiting microalgal blooms as a result of viral infections is emphasized.

## 1. Introduction

The search for new marine-derived molecules in previously insufficiently studied groups of marine organisms is one of the most important stages of drug discovery. Metabolites of microalgae are a promising source of such compounds. Microalgae (microphytes) are unicellular or colonial eukaryotic, mainly autotrophic organisms occurring in fresh, brackish, and seawaters from the surface layers down to the bottom sediments [1,2,3]. Diatoms (kingdom Chromista, phylum Ochrophyta) are an abundant and widely-distributed component of the marine phytoplankton, including over 8000 species [2]. A characteristic feature of diatoms is the porous frustule (cell wall) with two overlapping valves (epitheca and hypotheca) composed of silica. Some species of diatoms can produce a variety of natural products, including low-molecular-weight compounds [4]. The phylum Ochrophyta includes, along with diatoms, some other groups of marine, freshwater, and soil heterokontophytes (Eustigmatophyceae, Chrysophyceae, Chrysomerophyceae, Dictyochophyceae, Pelagophyceae, Raphidophyceae, Xanthophyceae, and others). Eustigmatophytes, coccoid microalgae inhabiting mainly freshwater habitats and soil, and so-called golden microalgae, belonging to the class Chrysophyceae, as well as species belonging to the related class Sinurophyceae with marine genera *Mallomonas* and *Synura*, sometimes produce toxins and other bioactive compounds [5]. Dinoflagellates (phylum Dinophyta, kingdom Chromista) represent another important component of phytoplankton, including about 2300 marine, freshwater, or parasitic species [2]. Dinophytes are abundant in warmer seas and in temperate areas during the warm seasons. They possess two dissimilar flagella and a cell cover composed of several membranes, containing polysaccharide plates (in armored dinoflagellates) [6]. Some species of diatoms and other heterokontophytes, as well as many dinoflagellates, can cause bloom events and produce dangerous toxins [6,7]. 

There are a series of other phyla of microalgae belonging to the kingdom Chromista. For example, the phylum Haptophyta (97 species) is an algal group with several classes, including very abundant in open ocean waters coccolithophorids (Coccolithophyceae) [1], which have an exoskeleton of calcareous plates (coccoliths). Coccolithophorids comprise the bloom-forming species belonging to the genera *Emiliania, Phaeocystis,* and *Chrysochromulina*, as well as the *Isochrysis* species, which are used in the oyster and shrimp aquaculture as food for larvae. Pavlovophyceae is another class of haptophytes whose metabolites have been extensively studied recently [8]. Cryptophytes (phylum Cryptophyta, kingdom Chromista) is a small group of marine and freshwater microalgae (about 200 species) which is abundant in brackish-water habitats and oligotrophic lakes. Some species live symbiotically inside ciliates, providing the host with photosynthates [3]. 

Glaucophytes (phylum Glaucophyta, approximately 15 species) are a very small group of rare freshwater microalgae, which, together with red and green algae, are combined into the kingdom Plantae [1]. Unicellular species of green (Chlorophyta, a total of ca. 8000 species) and red algae (Rhodophyta, ca. 7000 species) may be also referred to as microalgae. Euglenophytes (phylum Euglenozoa, ca. 2000 species) belong to the kingdom Protozoa [1,2], consisting mostly of unicellular motile microalgae. This algal group is abundant in freshwater habitats: puddles, ditches, ponds, streams, lakes, and rivers, particularly in waters exposed to pollutants or decaying organic matter [3].

Generally, most microalgae are capable of performing photosynthesis, and globally produce about a half of all organic substances and oxygen each year, although some groups are shown to be heterotrophs. All these lower plants lie at the base of food webs in the hydrosphere, providing the major part of matter and energy for higher trophic levels. Metabolites of microalgae play important roles in the lives of their producers, but also greatly influence other living organisms in the hydrosphere. 

Glycoconjugates and other conjugated forms are known as bioactive metabolites and signal compounds. Recently, new information appeared on the interesting biological properties of microalgal glycoconjugates. The aim of this first review in this scientific field is to summarize the current data concerning glycoconjugate chemical forms of sterols and sphingolipids from microalgae, which contain hydrophobic fragments of different biosynthetic origins with attached polar groups such as sugars. The review covers the period from 1978 to 2017.

## 2. Sterols and Sterol Glycoconjugates. Structural Diversity and Taxonomic Distribution

### 2.1. Free Sterols

Sterols of microalgae, as well as their fatty acids, were better examined in comparison with any other metabolites, but not in all the taxonomic groups of microalgae, and therefore, sterols in some taxa remain largely unstudied to date. Microalgal sterols are well known as the main source of steroidal materials of many other marine organisms, which obtain these compounds through their diet. The structural diversity of microalgal sterols and their biological significance were thoroughly reviewed [9,10,11,12,13]. One of the recognized experts in this scientific field, Volkman, recently wrote that “the published information on sterol compositions of microalgae continues to increase, but it is still far from comprehensive” [13]. 

**Figure 1:**
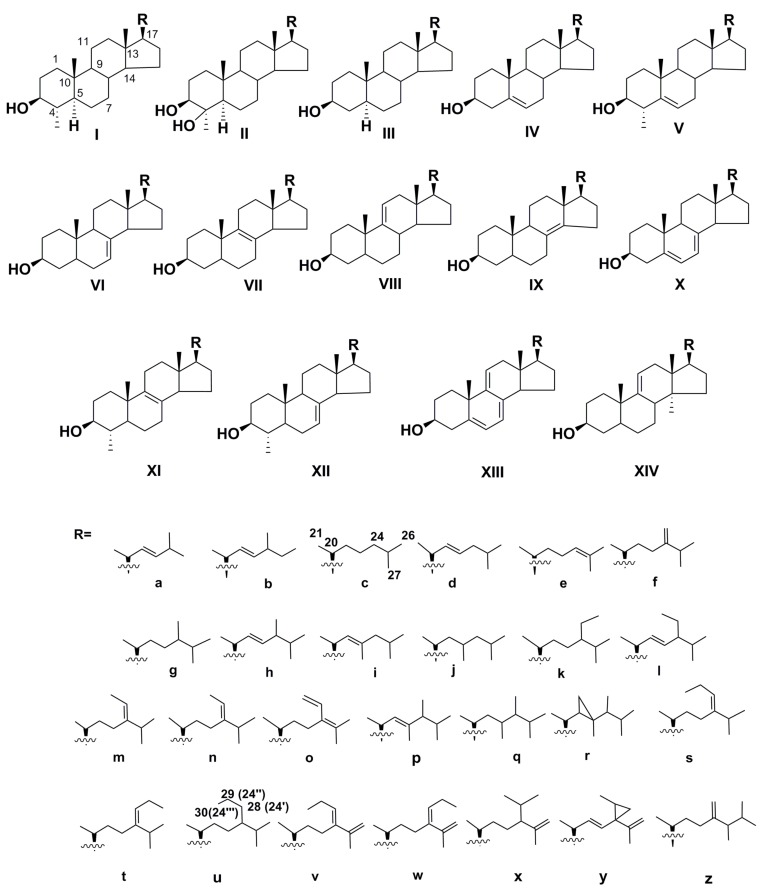
Sterols from microalgae.

Usually, the structural diversity of sterols is illustrated using the formulae of their tetracyclic nuclei differing from each other mainly in their unsaturation degree and positions of double bonds. These core fragments bear variable side chains. More than a dozen different tetracyclic cores and many variants of side chains were found in microalgal sterols, as shown in Figure 1. Significant variability in the corresponding fractions and their main constituents was established by comparing sterols of different taxonomic groups. Moreover, it was shown that many taxa contain unusual and rare sterols. A number of sterols of microalgae have not been found in terrestrial higher plants. 

For example, dinophytes are characterized by a great variety of unusual sterols, and often contain a saturated 4α-methyl-5α-cholestane type core, as in dinosterol (24*R*-Ip) and dinostanol (23*R*,24*R*-Iq). In Pelagophyceae, extremely rare C30 sterols such as 24-propylidenecholesterols (IVs,t) and other highly-alkylated in side chain compounds were found. Some other exotic sterols of microalgal origin are mentioned in Table 1, which shows the taxonomic distribution of sterols.

Frequently, the taxonomic distribution of sterols is analyzed on the class level, because the compositions of larger taxa, such as phyla, are still the subject of discussion and revisions by taxonomists. The structural diversity of sterols is great (Figure 1 and Table 1), and many of these compounds are discovered each year.

Generally, the diversity of numerous microalgal free sterols was found to be higher in comparison with that of higher plants. Sterol compositions of microalgae were discussed in numerous works, which considered the chemotaxonomic importance of these compounds, their application as markers for marine and terrigenous organic matter, their significance for marine life, and their influence on the development of their producers in wild conditions and cultures [9,10,11,12,13,20,26,27].

There is no doubt that microalgal sterols make a great contribution to steroid metabolism in marine animals, being an important food component of echinoderms, mollusks, and other invertebrates, and penetrating via food chains into fish and marine mammals. Another almost unstudied biological signification of these metabolites is associated with their potential participation in symbiotic relationships, because, as is well known, a tremendous number of marine organisms, particularly invertebrates, host microbial symbionts. Such membrane constituents as sterols in both micro- and macro-organisms might have co-evolved in these symbiotic consortiums. 

### 2.2. Structural Diversity and Analysis of Steryl Glycoconjugates

**Figure 2:**
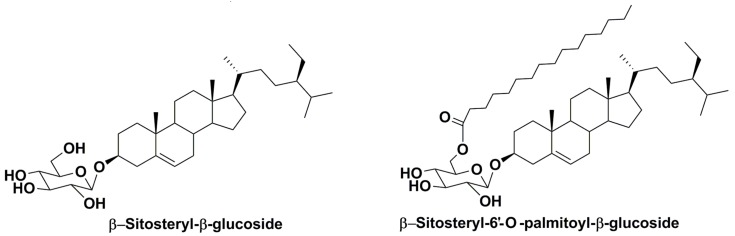
An example of glycoconjugated sterols from higher plants.

Sterols are present in microalgae in not only free, but also in conjugated forms. Actually, free sterols are frequently, if not always, accompanied in these microorganisms by conjugated forms such as steryl glycosides (SG), acyl steryl glycosides, esters (bearing fatty acid residues), and sulfates. Glycosylated forms of sterols in both higher and lower plants mainly include two types of natural compounds: glycosides of sterols and their acylated-by-fatty-acids derivatives. In these acylated glycosides, acylation proceeds, as considered, predominantly to the C-6 position of a sugar moiety (in majority cases glucose, but other monosaccharides may also be present), as shown below in the example of β-sitosterol glycoconjugates which are widely distributed in higher plants and are found in some microalgae (Figure 2).

Steryl glycoconjugates are known for their bioactivity (see Section 2.3 of this review). These metabolites have been found many times in nature, being isolated from different higher and lower plants, such as olives, soybeans, potatoes, and algae. Moreover, they are also abundant in many species of fungi, and have been detected in bacteria and animals. The concentration of these metabolites is commonly (but not always) almost one order of magnitude lower than that of free sterols. That is why these compounds were not detected in algae for a long time, and their absence was even considered a characteristic difference between higher plants and algae [28].

Frequently, information about these biochemical forms of sterols is lost in many studies on microalgal species, because researchers either hydrolyze extracts before isolation in order to determine the total sterol composition, or isolate only fractions of free sterols. In many papers, the authors established only the presence of a glycoconjugated fraction, without specifying its sterol profile, and provided the data on total sterol composition of all the sterol forms. For example, in a study on the heterocontophyte *Nannochloropsis* sp. (Eustigmatophyceae) and the fungoid organism *Schizochytrium limacinum* (superphylum Heteroconta, Thraustochytriaceae, Labyrinthulomycetes), which are promising for biofuel production, Wang and Wang [29] found fractions of steryl glucosides in both species, but did not establish their sterol profiles. They reported only the total sterol composition with main constituents IVc and IVm from *Nannochloropsis* and an unusual polyunsaturated sterol from *Schizochytrium*. However, later, the presence of this sterol was not confirmed [30].

As a rule, steryl glycosides from algae differ from those of higher plants in aglycone moieties. It was reported that isofucosteryl glucoside and clionasteryl glucoside are present in the macrophyte green algae *Ulva gigantea* and *Cladophora rupestris*, while fucosteryl glucoside and desmosteryl glucoside are characteristic metabolites of brown and red macrophytes *Fucus vesiculosus* and *Rhodymenia palmata*, respectively [31]. It was suggested that the sterol profile of steryl glycosides reflects the free sterol profile of the studied species, as confirmed by studies on terrestrial plants [32]. Moreover, first studies on microalgal steryl glycoconjugates from *Porphyridium* sp. also confirmed this suggestion [32].

However, subsequent investigations showed that it is not always true as regards microalgae. In fact, the microalgae *Tetraselmis chui* (Phylum Chlorophyta, Chlorodendrophyceae), *Nannochloropsis salina* (Phylum Ochrophyta, Eustigmatophyceae), and *Skeletonema costatum* (Phylum Ochrophyta, Bacillariophyceae), which are extensively used in mariculture, differ from each other in their free sterol compositions. The first of these species contained mainly 24-methylenecholesterol (IVf) and campesterol (24*R*-IVg) in free sterol fractions, the second, cholesterol (IVc) and its 22(23)-dehydroderivative (IVd); and *S. costatum*, at least four main sterols, including cholesterol (IVc), dehydrocholesterol (IVd), stigmasterol (IVl), and brassicasterol (24*S*-IVh). However, cholesterol was found as the only common fraction of free and all conjugated forms in these microalgae, although it is a minor constituent of free sterols in some of these species [33]. These results showed that the glycosylation may occur specifically in the studied microalgae, and that glycosylation leads to the formation of a variety of known and previously-unknown compounds. Thus, main glycoconjugated forms, when compared with free sterol fractions, may contain minor sterols as aglycones. 

Similar results were obtained by comparing free and conjugated sterols of *Pavlova lutheri* (Phylum Haptophyta, Pavlovophyceae). Uncommon 4α-methyl-24β-ethylcholest-22(23)-en-3β-ol (IIl) was identified as the dominant sterol constituent along with eight common desmethylsterols, including poriferasterol (24*R*-IVl) and cholesterol. In contrast to free sterol fraction, this dominant sterol was not found as aglycone in steryl glycosides, while glucosylated cholesterol and so-called pavlovols (3,4-dihydroxy-4α-methylsterol derivatives such as II c,d,f,g, Figure 1) were indicated as the main sterol constituents in glycosylated forms. Moreover, neither Il, nor pavlovols were identified in acetylated glycoconjugates with acylated cholesteryl glucoside as a main constituent. This shows that the acylation also occurs specifically, and cholesteryl glucoside is probably acetylated more effectively in comparison with other steryl glucosides in this species [34].

Later, a more detailed comparison of sterol compositions of free sterols, esterified sterol forms, steryl glucosides, and acylated steryl glucosides was carried out by the same group of researchers on seven unicellular algae [35]. The total sterol content varied from 91 μg/g dry weight in the diatom *Skeletonema costatum* to 1354 μg/g in *Isochrysis* aff. *galbana* (phylum Haptophyta, Isochrysidaceae). The latter species contained almost the same amount of conjugated and non-conjugated forms as *P. lutheri*, another haptophyte microalga studied earlier. It was of particular interest that free sterols were dominant not in all the studied species, but only in four of them: centric diatom *S. costatum*, haptophyte *Isochrysis* aff. *galbana*, chlorophyte *Tetraselmis suecica*, and pennate diatom *Haslea ostrearia.* At the same time, the eustigmatophyte *Nannochloropsis oculata* and centric diatom *Thalassiosira pseudonana* contained most of their sterols in esterified form, while the centric diatom *Chaetoceros calcitrans* had about 60% of its sterols in glycoconjugated form (as a sum of steryl glucosides and acylated steryl glucosides). Only *C. calcitrans* contained the same sterol (cholesterol) as the dominant constituent in conjugated and non-conjugated forms. Other studied microalgae had more or less different sterol compositions in free and glycoconjugated forms. For example, the diatom *H. ostrearia* contained mainly unusual 23,23-dimethylcholest-5-en-3β-ol in its glycoconjugated form.

In the pennate diatom *Phaeodactylum tricornutum*, a significant portion of sterols was found in glycosylated form with glucoside of 24-methylcholesta-5,22-dien-3β-ol as the main constituent. Levels and compositions of free and conjugated sterol forms in microalgae depend on culture conditions. The content of SG was 100-fold lower in *P. tricornutum* when this microalga was grown at 23 °C instead of 13 °C [36]. 

**Figure 3:**
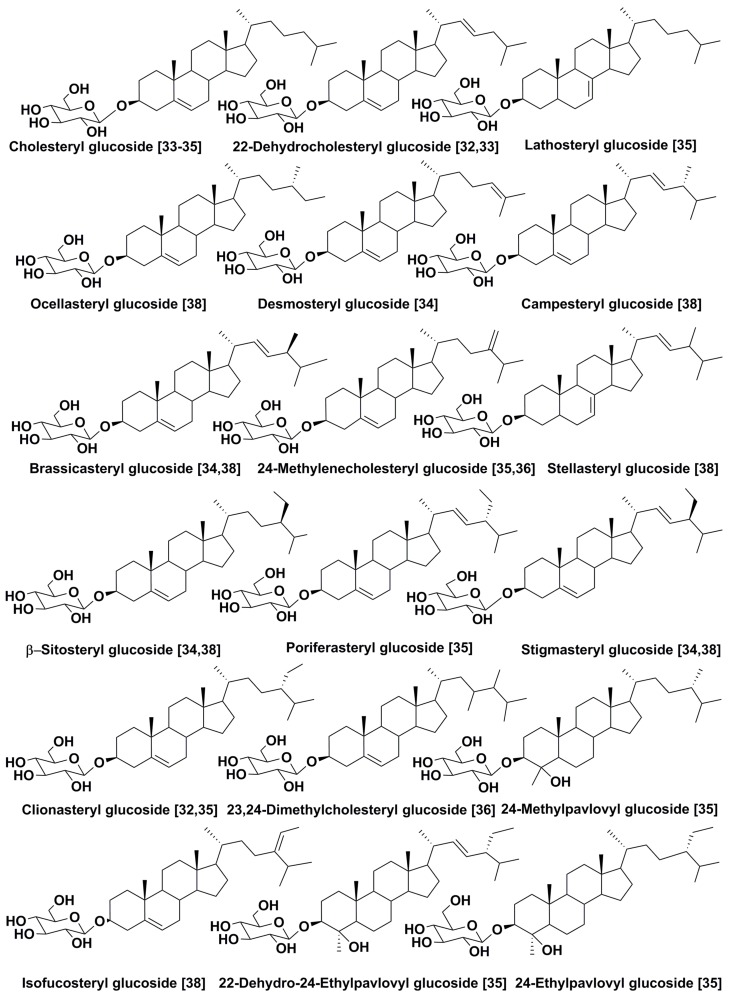
Steryl glucosides from microalgae.

Generally, glycoconjugated sterols in microalgae (Figure 3) are more poorly-studied compared to those in higher plants. However, even the limited available data on these metabolites in microalgae clearly show that the structural diversity of their steryl moieties is greater than that in higher plants (Figure 3).

Glycosides, including glucosides, glucoronides, galactosides, ribosides, and even compounds containing several monosaccharide residues, were found among steryl glycoconjugates of different origin [37]. In spite of this fact, all the thus far indentified glycoconjugates from microalgae, including those studied with application of mass-spectrometry, were found to contain only hexoses, probably D-glucose residues.

The main difficulty of a structural study on this class of natural compounds in microalgae is their low content and often the small amount of initial biomass, which could be used for their isolation and identification. The common approach to their identification includes the isolation of total glycosylated fraction using thin-layer or column chromatography on silica gel and application of NMR spectroscopy, that indicates the low-field shift of anomeric CH group signals and the appearance of characteristic signals of sugar moiety in comparison with spectra of free sterols. However, NMR spectroscopy has not yet been used in studies on microalgal steryl glycosides out of their low levels in biological sources. As a rule, acid and alkaline hydrolyses were carried out to obtain free sterols, and thereafter, to study them by GC or GC-mass-spectrometry (GS-MS) methods. 

The progress in this scientific field was stimulated by the development and application of more sensitive chromatographic and mass-spectrometric techniques, which made possible the identification and quantitative analysis of respective natural compounds in microgram quantities in some unstudied species. Also, new attempts have been made to find available sources of the respective glycosidases and esterases as biochemical tools for mild transformation of sterol glycoconjugates to free sterols. The additional interest in analysis of steryl glycoside content arose due to difficulties in obtaining biodiesel, associated with the steryl glycosides responsible for poor filterability of this product [38,39]. 

Solid-phase extraction followed by gas chromatography was proposed as a rapid and convenient method of analysis of steryl glycosides in foods and dietary supplements. For example, steryl glucosides, extracted with a hexane-diethyl ether (1:1, *v*/*v*) mixture after alkaline saponification of lipids, were isolated by solid-phase extraction, derivatized, and quantitively analyzed as trimethylsilyl ethers by capillary gas chromatography (GC) with a 5% diphenyl-95% dimethylpolysiloxane column [38]. 

A similar proposed variant of analysis involved the use of GS–MS of pretreated silylated samples, followed by single ion monitoring at 147, 204, 217 m/z, which are specific ions for the silylated sugar moiety [39].

Mass spectrometry combined with liquid chromatography also opens new possibilities and promises a rapid progress in identification of new variants of steryl glycosides [40]. 

**Figure 4:**
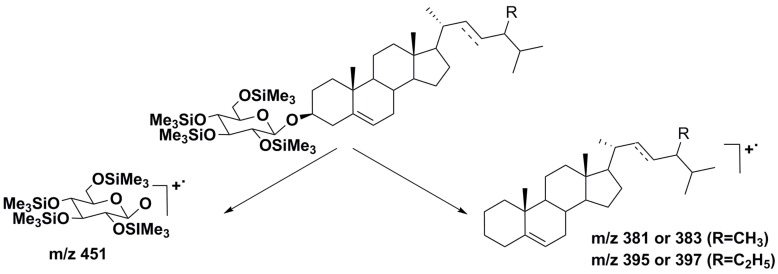
Fragmentation pathways of steryl glucosides in EI MS with the formation of either sugar or sterol cationic radical species.

Recently, a group of Chinese scientists used gas chromatography–triple quadrupole mass spectrometry to characterize steryl glycosides as their trimethyl silyl derivatives in eight microalgae [41]. As a result, not only nine compounds were identified, including several previously unknown glycosides, but also, characteristic fragmentations in the corresponding mass spectra were established. This method proved to be a powerful tool for finding new glycoconjugated sterols and promising new biological sources of highly valuable natural product substances in this class. The fragmentation pathways included the cleavage of a glycosidic bond that made it possible to identify ion peaks of both monosaccharide and sterol moieties in the studied conjugates (Figure 4).

Unfortunately, the application of solely chromatography-mass-spectrometry does not allow researchers to precisely identify the monosaccharide (or monosaccharides) present in glycoconjugates, nor to establish the type of glycoside bonds between sugars in di- or triglycosylated compounds, when these glycosides will be found. The additional use of NMR spectroscopy (including different 2D NMR techniques such as COSY, ROESY, HMBC and HSQC) or chemical transformations such as hydrolysis of permethylated derivative, followed by analysis of obtained sugar derivatives, is required in such cases. 

### 2.3. Biological Activities and Biological Functions

The most studied compounds of this class, e.g., β-sitosteryl and campesteryl glycosides from edible terrestrial higher plants and other biological sources, have attracted attention by their influence on immunological processes. An injection with sitosteryl glucoside increased the survival rate of mice infected by *Candida albicans*. A mixture of β-sitosterol and sitosteryl glycosides, so-called sitosterolin, increased the production IL-2 and IFN-γ cytokines by murine helper T cells stimulated by phytohemagglutinin. The immunomodulatory and protective effects of this preparation were used in treating patients suffering from allergic diseases. Daily administration of sitosterolin improved the health of patients with pulmonary tuberculosis and helped their recovery [42,43]. The fact that sitosterol and campesteryl glucosides are also produced by some microalgae is of particular interest (Figure 3).

Sterylglycosides with structural peculiarities in sugar moiety also showed noteworthy immunological properties. For example, the gram-negative bacterium *Helicobacter pylori*, colonizing the stomach and causing gastric diseases, is auxotrophic for cholesterol and extracts this lipid from plasma membranes of epithelial cells of the host stomach. This bacterium converts cholesterol into cholesteryl 3α-glucoside. Since the incorporation of cholesterol promotes immune responses of the host, *Helicobacter pylori* converts cholesterol to cholesteryl glucoside and then to the corresponding cholesteryl 6′-*O*-acyl glucoside, thus evading the immune surveillance. In another experiment, the analogous steryl β-galactoside caused a rise of antibodies against a *Borrelia burgdorferi* infection [43,44]. 

It was shown that phytosteryl glucosides, separated from the crude soybean lecithin and solubilized in purified soybean oil, influenced the cholesterol adsorption. When plasma cholesterol of patients after consumption of a test breakfast with pudding containing lecithin and cholesterol was analyzed, it was highest after the placebo test. However, the LDL cholesterol absorption in eleven patients 4 and 5 days later was reduced by almost 40% after the addition of phytosterol glucosides to their diet [45]. It was shown that acylated steryl glycosides also reduce cholesterol absorption in mice as efficiently as phytosteryl esters. Cleavage of the glycosidic bond in steryl glycosides is not required for their biological activity. Moreover, these bonds are not cleaved in animals which consume food containing these compounds [46].

The nutritional value of food supplements derived from chlorophytes *Chlorella*, *Scenedesmus*, *Nannochloropsis,* and *Dunaliella* is well known [47]. Generally, phytosterol-containing products, including phytosterols as well as their glucosides and acylated glucosides (phytosterolins), have been commercialized as nutriceuticals or pharmaceuticals capable of lowering the blood cholesterol level and preventing the onset of cardiovascular disorders. The administration of these agents and a proper diet, enriched in fruits and vegetables, improves immune status and may help in treating various health problems related to chronic immune-mediated abnormalities. Microalgae can be considered as a promising source of these valuable compounds. The sterol content of such microalgal species as the haptophyte *P. lutheri*, chlorophytes *Tetraselmis* sp. and *Nannochloropsis* sp. was reported to range from 0.4–2.6% of dry weight. Taking into consideration their fast-growing characteristics, the annual production of fatty acid- and sterol-enriched oil from microalgae may vary from 19,000 to 57,000 L, which is greater than that obtained from terrestrial higher plants [48]. 

The great nutritional value of microalgae is first of all associated with the accumulation of polyunsaturated fatty acids in their lipids. The study on lipid classes of the haptophyte *P. lutheri* showed that acylated steryl glucosides in this species contain mainly C16:1(n-7), C14:0, and C16:0 fatty acids, but with a much higher level of docosahexaenic acid than that of eicosapentaenic acid in minor fatty acids [49]. Thus, acylated steryl glycosides of food supplements from microalgae also provide some contribution not only to the total phytosterol content, but also to the pool of useful polyunsaturated acids, characteristic of microalgae.

Steryl glycosides are also involved in cellular stress response. The level of sterol glycosylation is graduated in microalgae exposed to heat shock [36]. 

**Figure 5:**
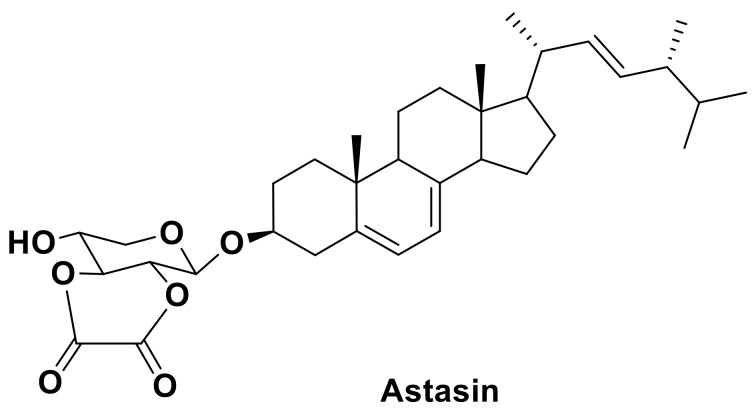
Unusual steryl glycoside astasin.

Steryl glycosides of unusual structures, possessing by other biological properties, can be also found in microalgae. For example, astasin, an unusual cytotoxic modified steryl glycoside from the colorless euglenophyte *Astasia longa*, consisting of ergosterol, xylopyranose and oxalic acid (Figure 5), has become an unexpectedly valuable find [50]. This compound inhibits the growth of human lymphoma HL-60 cells. 

Biological functions of glycosylated sterols are most probably associated with the alteration of biophysical properties of cell membranes by these compounds. It was established that cholesteryl glycosides were much less effective in comparison with cholesterol in ordering the hydrocarbon chain region in the sphigolipid bilayer [51]. Steryl glycosides, which are amongst the main plant membrane components, were supposed to regulate the action of hormonal and environmental signals, thus providing the organization and biophysical properties of these membranes [52,53]. The consequences of variations in the proportions of free and glycosylated sterols in membranes are still not quite clear; however, the absence or shortage of steryl glycosides leads to dramatic dysfunctions in their producers [52]. Nevertheless, to the best of our knowledge, the biological functions of sterylglycosides on a molecular level and their effects on the growth and development in microalgae remain insufficiently studied so far.

Recently, it was found that an additional biological function of steryl glycosides possibly consists of their participation in biosynthesis of polysaccharides. In plants, cellulose synthase initiates the glucan synthesis using sitosterol-β-d-glucoside as primer. β-Sitosterol cellodextrins are formed from sitosterol-β-d-glucoside and uridine-5’-diphosphate glucose under conditions favorable for cellulose synthesis [54]. However, it is unclear whether this function is implemented in lower plants such as microalgae. 

### 2.4. Biosynthesis of Glycosylated Sterols

The biosynthesis of steryl glycosides in plants, including some microalgae, occurs with the participation of sterol glycosyltransferases and uridine diphosphate glucose (UDP) as a sugar donor; this was shown using the example of the parasitic chlorophyte *Protothecha zopfii* [55]. As suggested, 4-methyl and 4,4-dimethyl sterols are poor substrates for these glycosylating enzymes [56]. Nevertheless, several glycosides of 4α-methylated sterols were detected in microalgae, which contradicts this suggestion (see Figure 3).

The membrane-bound sterol glycosytransferases (UDP-sugar:sterol glycopyranosyltransferases) belong to the family 1 of glycosyltransferases (UGT-superfamily) and hold an important position in plant metabolism [57,58]. The recent cloning of sterol glycosyltransferase genes from higher plants, algae, fungi, and bacteria was used to analyze the steryl glycoside functions. The corresponding full-length amino acid sequences of diatoms *Phaeodactylum tricornutum* and *Thalassiosira pseudonana*, as well as the sequences of the chlorophyte *Chlamydomonas reinhardtii*, were compared with those of related enzymes, and were functionally identified as sterol β-glycosyltransferases, deduced from genomic DNA. It was shown that the down regulation of sterol glycoside biosynthesis in the higher plant *Arabidopsis thaliana* causes dysfunctions in seed development [52]. 

**Figure 6:**
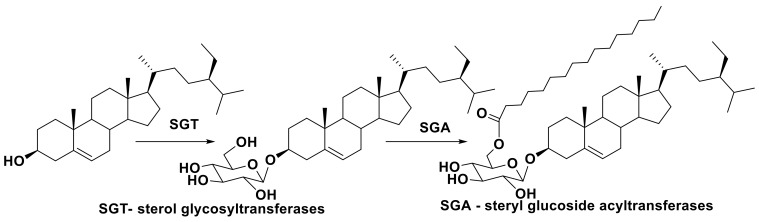
Biosynthesis of sterol glycoconjugates.

Sterol glycoconjugates, acylated at C-6 of sugar portion, found in many plants including microalgae, are biosynthesized with the participation of another group of enzymes, namely steryl glycoside:acyltransferases [59]. A scheme of biosynthesis of steryl glycoconjugates, using glycosylated forms of sitosterol as an example, is given in Figure 6.

## 3. Sphingoid Glycoconjugates

Sphingolipids, found in almost all animals, plants, and fungi, as well as in some prokaryotic organisms and viruses, are an important part of the corresponding lipidomes. These compounds perform important structural and intracellular functions and participate in extracellular signaling. Along with sterols, they form the specialized microdomains in plasma membranes which are involved in a great variety of cellular processes, and are known as “rafts” [60]. 

**Figure 7:**
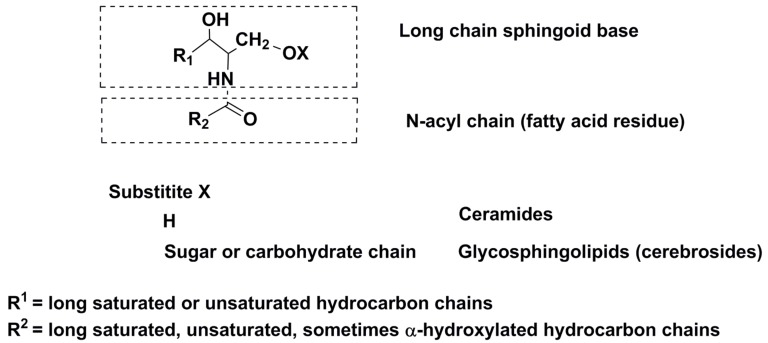
General structures of some sphingolipids (ceramides and glycosphingolipids).

Glycosphingolipids (Figure 7), as a class of sphingolipids, differ from non-glycosylated forms (ceramides) by a greater structural diversity: in addition to amine-containing lipid backbones, consisting of so-called sphingoid bases (sphingosine, sphinganine, phytosphinganine, and others) and fatty acid residues, they may contain a variety monosaccharide or oligosaccharide moieties. Moreover, these metabolites are sometimes sulfated and rarely phosphorylated [61].

The structural diversity of glycosphingolipids from mammals, higher plants, and fungi has been extensively studied for many years. A significant difference has been shown to exist between these metabolites from different taxa, particularly in their ceramide backbones. However, lower plants remain poorly studied in this respect to date. Many of marine animal species ingest microalgal metabolites obtained via the food chain. Moreover, there are many marine invertebrates containing microalgal symbionts. These host-symbiont consortiums produce a variety of highly bioactive natural compounds such as glycosphingolipids, biosynthesizing most probably with the participation of these symbiotic microorganisms. For example, the obligate symbiotic relationship between cnidarians such as corals and some dinoflagellates of the family Symbiodiniaceae is well known [62]. A comparison of their sphingolipids with those of whole symbiotic complexes would be of interest to better understand the peculiarities of the biosynthesis of these compounds in the plant and animal kingdoms. 

### 3.1. Structural Diversity

**Figure 8:**
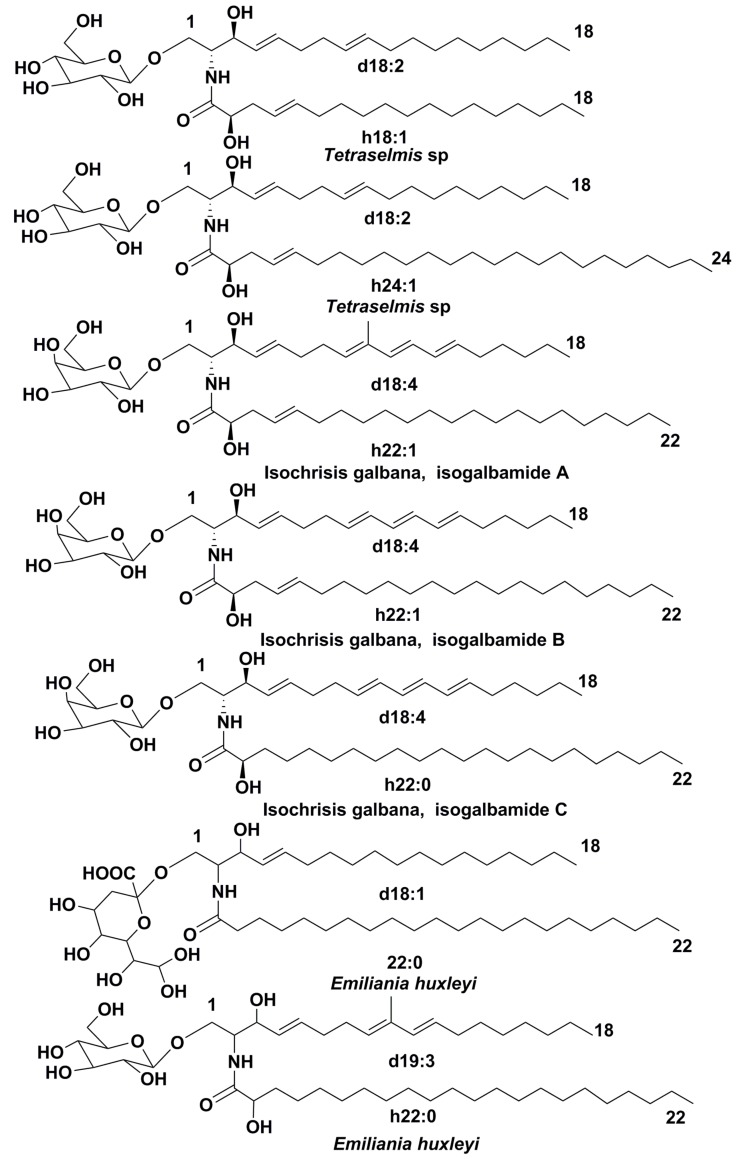
Glycosylceramides from the microalgae *Tetraselmis* sp., *Isochrisis galbana*, and *Emiliania huxleyi.* Sphingoid base is designated with the chain length and number of double bonds; prefix ‘d ’ is used to designate dihydroxylated bases. Fatty acids residues are designated with the chain length and number of double bonds; prefix ‘h’ is used for hydroxylated fatty acids.

Glycosylceramides (GlyCer) were detected in a large number of microalgae species, but in most of these cases, their structures were not fully characterized. As a rule, the determination of positions and configurations of double bonds, as well as the exact identification of sugars, were not performed. The first structural study on glycosylceramides from marine microalgae belonging to the class Prasinophyceae was carried out by Japanese scientists, who isolated two unusual glycolipids from *Tetraselmis* sp. (Figure 8). The chemical structures of these compounds were determined by NMR spectroscopy and GC-MS. Their sphingosine bases have (4*E*,8*E*)-sphinga-4,8-dienic structures (d18:2) and resemble the related lipids from terrestrial higher plants, while fatty acid moieties consist of 2-hydroxy-Δ^3^-unsaturated fatty acid residues (h18:1 and h24:1). Δ^3^(*E*)-unsaturation appears to be earlier found in some fungal glycosylceramides, but all these metabolites from fungi contain a characteristic C9-methyl branching in sphingoid moieties. Moreover, such long fatty acyl chains as C24 are rarely identified in fungal cerebrosides. Thus, the isolated metabolites are representatives of previously unknown structural series [63]. 

Three new glycosylceramides named isogalbamides A–C were isolated from the microalga *Isochrysis galbana* (Haptophyta, class Coccolithophyceae) using silica gel column chromatography and reversed phase HPLC, and studied by NMR and MS/MS methods. These first galactose-containing microalgal glycoconjugates have unprecedented tetraunsaturated sphigoid bases with structures of monosaccharide, N-acyl chains and bases, confirmed by COSY, HSQC, and HMBS NMR spectra. Isogalbamide A contains methyl branching at C-9 in the sphingoid base, like some fungal glycosylceramides, but closely-related isogalbamides C and D bear normal chains in these moieties. Isogalbamides showed moderate activity as inhibitors of the production of pro-inflammatory cytokine TNF-α in lipopolysaccharide-stimulated human THP-1 macrophages [64]. The procedures of extraction, separation, and spectroscopic analysis provided in this paper will be significant for forthcoming studies on microalgal glycosylceramides.

The identification of GlyCer with dC18:2 long chain base and 2-keto-3-deoxynonic acid (Kdn) as monosaccharide, as well the corresponding compound with methylated long-chain base, acylated by C22:0, C22:1, C22:2 and C-22:3 fatty acids, from the *Emiliana huxleyi* (phylum Haptophyta, Coccolithophyceae) has become another unexpectedly valuable finding (Figure 8) [65].

The diatom *Skeletonema costatum* was also recently examined for this type of important membrane component. A separation of lipids, extracted from this alga, using reverse-phase liquid chromatography, provided a fraction, which was subsequently studied by tandem mass spectrometry with collision–induced dissociation of the lithiated adducts using electrospray ionization quadrupole time-of-flight mass analyzer. As result, many types of novel glycosphingolipids were characterized from three strains of this alga, although their exact structures were not completely established. One of these strains (SCXMB02) contained disaccharides type of the corresponding compounds with 12 variants of mono- and diunsaturated C18 bases. N-acyl groups were primarily saturated or monoenic with 16C and 20 to 24C. The polar head groups were disaccharides consisting of heptose and hexose. Another strain (SKPXS0711) contained 13 related disaccharide ceramides, including those with triunsaturated long-chain bases. 

The third strain (SKSPXs0807zjj) had novel glycosphingolipids of another type with trisaccharide moiety (heptose-hexose-hexose). This strain was considered as another species of the genus *Skeletonema* [66,67]. It is of particular interest that no trace of hexosylceramides and lactosylceramides, which are characteristic of higher plants, was found in the studied strains of *Skeletonema.* N-acyl groups of these glycosphingolipids were non-hydroxylated fatty acids, in contrast with the corresponding metabolites of higher plants. Thus, these studies demonstrated a great diversity of novel sphingoid glycoconjugates in diatoms. 

Moreover, glycosphingolipid compositions of 17 microalgal strains belonging to species of the phyla Ochrophyta (2 strains of *Skeletonema costatum* and one strain of each *Skeletonema* sp., *S. tropicum*, *Thalassiosira pseudonana*, *Stephanodiscus* sp., *Conticribra weissflogii*, *Ceratoneis closterium*, *Ceratoneis* sp., *Phaeodactylum tricornutum*, and *Amphora* sp.), Dinophyta (*Alexandrium minitum*, *Prorocentrum donghaiense*, and *Karlodinium veneficum*), and Haptophyta (*Isochrysis galbana*, *I. zhanjiangensis*, and *Pleurochrysis carterae*) have been also studied. A total of more than 40 variants of glycosylated ceramides have been detected in these strains [68]. As a result, the studied microalgae showed a great diversity of glyconjugated compounds (Table 2). 

It has been found that the content of these compounds depends on the conditions of their cultivation. In the diatom *T. pseudonana* grown in the phosphorus-limited conditions, the level of diglycosylceramides increased by up to ten-fold. Glycosphingolipids in this species were identified as new representatives of diglycosylated compounds with the main (Gly)_2_Cer d18:3/24:0 constituent [69]. Some results of glycosphingolipid identification and their taxonomic distribution are given in Table 2.

The obtained results showed the structural diversity of these metabolites in microalgae: the identified glycosphingolipids contained various long chain bases, a set of different non-hydroxylated and α-hydroxylated fatty acids, and from one to three monosaccharide units in their carbohydrate moieties. Despite the fact that the exact structures of glycosphingolipids have been determined only in a few cases, it may be expected that many novel structural variants of these glycoconjugates should be discovered in the near future.

### 3.2. Biosynthesis, Biological Activities and Biological Roles of Microalgal Glycosphingolipids

**Figure 9:**
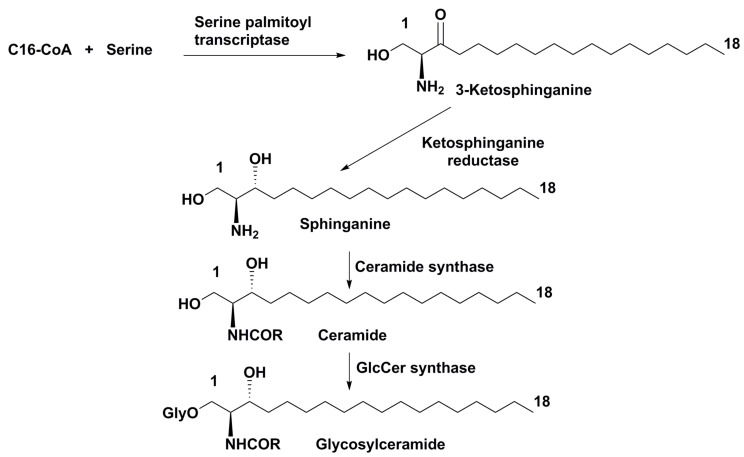
The main pathway of glycosylceramide biosynthesis.

Structures and taxonomic distribution of glycosphingolipids suggest that biosynthesis of these metabolites proceeds in general terms as in other plants. However, in different micoalgal taxa, there are numerous peculiarities and deviations that will undoubtedly be the subject of further research. The main pathway of glycosylceramide biosynthesis is shown in the Figure 9 [70]. However, not one compound, but whole sets of metabolites can be formed at each its stage depending from organism producer. For example, sphinganine may be transformed into 4-hydroxysphinganine as a result of 4-hydroxylation in some plants and marine organisms, and long chain bases may be formed as a result of sphinganine degradation followed by other transformations. Ceramide synthesis in some cases is accompanied by hydroxylation of fatty acid residues, giving a pool of different ceramides. Their glycosylation is sometimes not limited by the attachment of one monosaccharide unit, and so on.

Glycosylceramides, as ubiquitous glycosphingolipids occurring in plants, fungi, and animals, proved to be major components of the membranes in most eukaryotic cells. There is little information on the functions of individual representatives of these membrane constituents in both higher and lower plants. It was shown that glycolipids in membrane regulate some intracellular processes. The GlyCer content of the plant plasmatic membrane was observed to decrease in stress conditions such as, for example, cold acclimation. In these cases, the compositions of glycosphingolipids in microalgae are also changed [71].

It was established that GlcCer participate in cell-to-cell interactions [69]. These compounds may facilitate binding of pathogens to cells of the host, as with e.g., the human pathogen *Helicobacter pylori* binds monoglucosylceramides [72]. 

An important recent discovery is the participation of glycosphingolipids in the regulation of cell death caused by a viral infection in marine phytoplankton. The basis for this study was created by the complete genome sequencing of recently-discovered *Coccolithovirus*, the giant double-stranded DNA virus encoding approximately 600 proteins. Among the genes of this virus, a variety of previously unknown genes was found, including those involved in biosynthesis of sphingolipids [73]. 

The virus infects the coccolithophorid *E. huxleyi*, a cosmopolitan microalga, accounting for approximately a third of the global marine CaCO_3_ production due to its calcite skeleton and emission of dimethyl sulfide, which has an influence on cloud formation. The extensive annual rapid development into short-term large populations (blooms) of this species in the North Atlantic are terminated by the viral infection resulting in the lysis of microalgal cells. The genome of this type of viruses penetrates into host cells and changes the metabolism of glycosphingolipids during infection. Particularly, the virus EhV86 encodes the serine palmitoyl transferase (SPT), which induces a metabolic switch in sphingolipid biosynthesis in infected microalga, modulating the algal physiology. Viral glycosphingolipids, when interacting with cells of the microalga, suppressed the cell growth, elevated in vivo caspase activities, and caused mortality of the cells, inducing their apoptosis [74]. Composed of unique hydroxylated sphingoid base (t17:0), viral glycosphingolipids not only penetrate into microalgae, being essential for the infection process, but also participate in the virus assembly formation.

**Figure 10:**
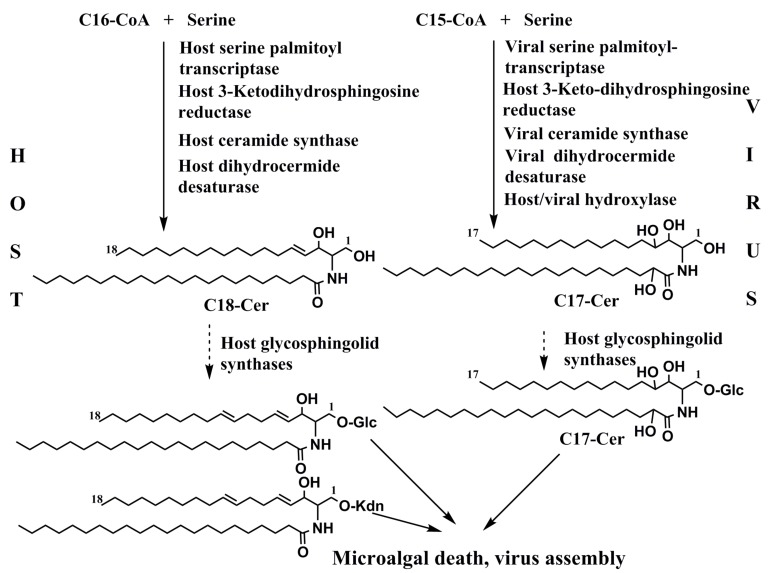
A simplified scheme of glycosylceramide biosynthetic pathways during interaction between the coccolithophorid *E. huxleyi* and its specific virus ExV.

An abridged scheme of participation of the virus in sphingolipid biosynthesis during the viral infection in the coccolithophorid *E. huxleyi* is provided in Figure 10. The viral-encoded serine palmitoyl transferase (SPT), in comparison with this enzyme of the host, has a different substrate specificity, which allows C15-CoA instead of C16-CoA to produce the C17 sphinganine base instead of C18 sphinganine in the host cells. Further transformations, including glycosylation and hydroxylation, lead to a metabolic remodulation in sphingolipid biosynthesis. A viral SPT proved to be a key enzyme in rewiring the host ceramide synthesis [75]. 

The critical role of glycosphingolipids in the stimulation of programmed cell death of *E. huxleyi* was confirmed in the process of its natural bloom event. These metabolites also contribute to understanding the co-evolutionary “arm race” between populations of *E. huxleyi* and cocolithoviruses [76]. 

Some other conjugated lipids also were recognized as regulators of algal death at final stages of microalgal blooms. Actually, sterol sulfates were proved to be regulatory molecules of a cell death program in *Skeletonema marinoi*, a marine diatom bloom-forming species inhabiting temperate coastal waters. Intracellular level of sterol sulfates increases with cell ageing, that leads to an oxidative burst and the production of nitric oxide followed by apoptosis-like death of these microalgae [77].

An assumption that glycosphingolipids and sterol glycosides can be biogenetically related to each other is interesting. There is some evidence that glycosylceramides can be partly synthesized in plants through the interaction of ceramides with steryl glucosides as glucose donors [78,79,80]. However, this transformation has not yet been confirmed in microalgae. 

## 4. Conclusions

Glycoconjugates of sterols and sphingolipids are widely distributed metabolites of microalgae, although they are still insufficiently studied on the structural level in many taxa. Glycosylated sterols play important functional roles in their producers and exhibit different bioactivities, making them promising agents for applications in medicine and as supplementary components to healthy food. Sterols and their conjugates enter marine invertebrates from microalgae through the food chain and may be transformed into other steroid derivatives such as 7(8)-unsaturated compounds in many starfishes and sea cucumbers, or polyhydroxylated sterols and glycosides in starfishes and some other marine invertebrates. The corresponding metabolites from invertebrates often have the same structural peculiarities of their side chains as those of microalgal sterols and their conjugates [81,82], although the microalgal source of some invertebrate sterols, for example, containing a side chain (a) (Figure 1), has not yet been confirmed. 

Glycosphingolipids have been intensively studied in animals for decades, but knowledge of these metabolites in microalgae still remains poor. Recently microbial metabolites of this class have been successfully studied using modern variants of mass-spectrometry and chromatography. 

It was found that glycosphingolipids and sterol conjugates are regulators of membrane functions. Some of them are important for the interactions of their producers with pathogens, and may induce apoptosis in microalgae. Also, these compounds participate in the termination of microalgal blooms. 

## Figures and Tables

**Table 1:** Distribution of sterols in some taxa of microalgae.

Taxa	Sterols
**Ochrophyta**	
Bacillariophyceae	C_28_Δ^5,24(28)^ (IVf), 24*R*-C_28_Δ^5,22^ (epibrassicasterol IVh) [10,11,12,13], C_27_Δ^5^ (IVc), 27-nor-C_28_Δ^5,22^ (ocellasterol IVb), C_29_Δ^5,22^ (IVl [11,13], C_29_Δ^5^ (IVk) [14], previously unknown C_28_Δ^7,22^ (VIi), C_27_Δ^5,22^ (IVd) [13], C_29_Δ^22^ (IIIp), C_30_Δ^22^ (dinosterol Ip), cyclopropane sterols (Ir, gorgosterol IVr [15,16], C_28_Δ^7,22^ (VIh), C_28_Δ^8(9)^ (VIIg), C_29_Δ^24,28^ (IVo and IIIo) [13].
Eustigmatophyceae	C_28_Δ^5^ (IVg), C_29_Δ^5,24(28)^ (IVn), C_29_Δ^5,24(28)^ (IVm), C_29_Δ^5^ (IVk), C_28_Δ^5,24(28)^ (IVf), C_27_Δ^5^ (IVc) [12,13].
Pelagophyceae	C_30_Δ^5^ (IVu), C_30_Δ^5,24(28)^ (IVs,t), rare C_30_Δ^5,24(28),25(26)^ (IVv,w), trace C_30_ sterols (IVx-z) [13,17]
Chrysophyceae	24*S*- and 24*R*-C_29_Δ^5,22^ (IVl), 24*S*-C_28_Δ^5,22^ (IVh), C_28_Δ^5,7,22^ (Xh), 24*R*- and 24*S*-C_29_Δ^5^ (IVk), C_29_Δ^5,7,22^ (Xl), C_29_Δ^5,24(28)^ (IVm,n), C_29_Δ^7,24(28)^ (Xm),
Synurophyceae	C_27_Δ_5_ (IVc), C_29_Δ^5^ (IVk) [13]
Chrysomerophyceae	C_29_Δ^5^ (IVk), C_28_Δ^5,24(28)^ (IVf), C_27_Δ^5^ (IVc), C_29_Δ^5,22^ (IVl), C_29_Δ^5,24(28)^ (IVn), C_28_Δ^5,22^ (IVh) [13]
Xanthophyceae	C_27_Δ_5_ (IVc), C_29_Δ^5^ (IVk) [13]
Dictyophyceae	C_28_Δ^5,24(28)^ (IVf), C_28_Δ^5,22^ (Vd) [13]
Rhaphidophyceae	24*S*- and 24*R*-C_29_Δ^5^ (IVk), C_27_Δ^5^ (IVc), C_27_Δ^8(9)^ (VIIc), C_29_Δ^5,22^ (IVl), 27-nor-C_27_Δ^5,22^ (IVb), C_29_Δ^0^ (IIIk) [13,18]
**Dinophyta**	
Dinophyceae	C_30_Δ^22^ (Ip dinosterol), C_29_Δ^0^ (Iq), C_29_Δ^0^ (1g), C_29_Δ^24(28)^(If), C_28_Δ^22^ (IIIh), C_29_Δ^22^ (IIIp), C_28_Δ^8(14)^ (IXc), C_28_Δ^8(14),24(28)^ (amphisterol IXf), C_28_Δ^8(14),22^ (IXh), C_27_Δ^8(14),22^ (IXb), C_28_Δ^8(14),22^ (IXi) and others [13,19,20]
**Cryptophyta**	
Cryptophyceae	C_28_Δ^5,22^ (IVh), C_27_Δ^5^ (IVc), C_29_Δ^5,22^ (IVl) [13,18]
**Haptophyta**	
Coccolithophyceae	C_28_Δ^5,22^ (*epi*brassicasterol IVh), C_29_Δ^5,22^ (stigmasterol IVl), C_27_Δ_5_ (IVc), C_28_Δ^5,24(28)^ (IVf) [13]
Pavlovophyceae	C_29_Δ^5,22^ (IVl), C_27_Δ^5^ (IVc), C_29_Δ^22^ (IIIl), C_30_Δ^22^ (Il), pavlovols IIg, IIk,IId, minor Ig,h [13,21,22]
**Euglenophyta**	
Euglenophyceae	C_28_Δ^5,7,22^ (ergosterol Xh), C_29_Δ^8(9)^ (XIg), C_28_Δ^5,7,24(28)^ (Xf), C_29_Δ^5,7^ (Xk), C_27_Δ^5^ (IVc), C_29_Δ^5,22^ (IVl), C_28_Δ^5^ (IVg), C_28_Δ^5,22^ (IVh), C_27_Δ^0^ (IIIc), 23-unsaturated C_29_Δ^5,7^ (Xk) [13]
**Glaucophyta**	
Glaucophyceae	C_28_Δ^5,24(28)^ (IVf), C_29_Δ^5,22^ (IVl), C_29_Δ^5^ (IVk) [13,23]
**Cercozoa**	
Chlorarachniophyceae	C_28_Δ^5,22^ (IVh), C_29_Δ^5,22^ (IVl) [24]
**Rhodophyta**	
Porphyridiophyceae	C_28_Δ^5,7,22^ (ergosterol Xh), C_27_Δ^5,22^ (IVd), C_28_Δ^8,22^ (XId), C_29_Δ^8,22^ (XIh), C_28_Δ^8^ (VIIg), C_28_Δ^8^ (XIc) [13]
Stylonematophyceae	C_28_Δ^5,22^ (IVh), C_27_Δ^5^ (IVc), C_28_Δ^7^ (XIIc), C_28_Δ^7,22^ (XIId) [13]
**Chlorophyta**	
Prasinophyceae	C_29_Δ^5,24(28)^ (IVm), C_29_Δ^5,24(28)^ (IVn) [24], C_28_Δ^5,24(28)^ (IVf), C_29_^,5,7,22^ (Xl), rare C_28_Δ^5,7,9(11),22^ (XIIIh), C_29_Δ^5,7,9(11),22^ (XIIIl) [10,13]
Chlorophyceae	C_27_Δ^5^ (IVc), C_28_Δ^5^ (IVg), C_29_Δ^5,22^ (IVl) C_29_Δ^7,22^ (VId), C_28_Δ^7^ (VIg) [13,25,26]
Trebouxiophyceae	C_27_Δ^5^ (IVc), 24S-C_29_Δ^5^ (clionasterol IVk), C_29_Δ^5,22^ (poriferasterol IVl), C_28_Δ^5^ (IVg), C_29_Δ^5,7,22^ (7-dehydroporiferasterol Xl), C_29_Δ^7,22^ (chondrillasterol VIl), C_28_Δ^8^ (VIIg), C_28_Δ^8,22^ (VIIh), C_28_Δ^5,7,22^ (ergosterol Xh), unusual Δ^9(11)^-sterols: C_28_Δ^9(11)^ (VIIIg), C_29_Δ^9(11)^ (XIVg), C_28_Δ^5^^5,7,9(11),22^ (XIIIh), C_29_Δ^5,7,9(11),22^ (XIIIl) [13]
Chlorodendrophyceae	C_28_Δ^5,24(28)^ (IVf), C_28_Δ^5^ (IVg), C_27_Δ^5^ (IVc), C_27_Δ^5,22^ (IVd), C_27_Δ^5,24^ (IVe) [13]
	C_29_Δ^5,22^ (stigmasterol IVl), C_28_Δ^5^ (IVg) [13]

**Table 2:** Distribution of some structural types of glycosphingolipids in major taxa of marine microalgae.

Glycosphingolipids	Taxa		
	Ochrophyta	Dinophyta	Haptophyta
Ceramide moiety	d18:0/16:0; d18:1/16:0; d18:2/16:0; d18:1/22:0; d18:2/22:0; d18:3/22:0; d18:2/22:1; d18:3/23:0; d18:1/24:0; d18:2/24:0; d18:3/24:0; 18:2/24:1; d18:3/24:1; d18:2/24:2 d18:1/26:0;d18:2/26:0 [66,67]; d18:2/14:0; d18:3/14:0 [68]	d18:3/16:0; d18:4/16:0; d18:3/16:1; d18:4/16:1; d19:3/16:0; 19:4/16:1; d19:3/h18:1; 19:3/h19:1; d19:3/h24:1; d19:4/h24:1 [68]	d18:2^4,8^/h18:1^4^; d18:1^4,8^/h24:1^4^ [63]; d18:4^4,8,10,12^/h22:1^4^; 9-methyl-d18:4^4,8,10,12^/h22:1^4^; d18:4^4,8,10,12^/h22:1^4^; d18:1^4,8,10,12^ /h22:0 [64]; h18:1^4^/h22:0; C19:3^4,8,10^/d22:0 [65]; d18:0/h22:0; d18:0/ h22:1; d18:0/h22:2, d19:2/h22:0; d19:2/h22:1; d19:2/h22:2; d18:3/h23:2 and others [68]
Glycosyl moiety	Monosaccharide Disaccharide Trisaccharide	Monosaccharide	Glucose [63], galactose [64], glucose and sialic acid [65]
